# Spatiotemporal variations in exposure: Chagas disease in Colombia as a case study

**DOI:** 10.1186/s12874-021-01477-6

**Published:** 2022-01-13

**Authors:** Julia Ledien, Zulma M. Cucunubá, Gabriel Parra-Henao, Eliana Rodríguez-Monguí, Andrew P. Dobson, María-Gloria Basáñez, Pierre Nouvellet

**Affiliations:** 1 School of Life Sciences, University of Sussex, Brighton, UK; 2 London Centre for Neglected Tropical Disease Research & MRC Centre for Global Infectious Disease Analysis, Imperial College London, London, UK; 3 Departamento de Epidemiología Clínica y Bioestadística, Facultad de Medicina, Universidad Javeriana, Bogotá, Colombia; 4 Centro de Investigación en Salud para el Trópico, Universidad Cooperativa de Colombia, Santa Marta, Colombia; 5 National Institute of Health, Bogotá, Colombia; 6 Neglected, Tropical and Vector Borne Diseases Program, Pan American Health Organization (PAHO), Bogotá, Colombia; 7 Ecology & Evolutionary Biology, Princeton University, Princeton, USA

**Keywords:** Force of infection, Model averaging, Chagas disease, Infectious disease, Modelling

## Abstract

Age-stratified serosurvey data are often used to understand spatiotemporal trends in disease incidence and exposure through estimating the Force-of-Infection (FoI). Typically, median or mean FoI estimates are used as the response variable in predictive models, often overlooking the uncertainty in estimated FoI values when fitting models and evaluating their predictive ability. To assess how this uncertainty impact predictions, we compared three approaches with three levels of uncertainty integration. We propose a performance indicator to assess how predictions reflect initial uncertainty.

In Colombia, 76 serosurveys (1980–2014) conducted at municipality level provided age-stratified Chagas disease prevalence data. The yearly FoI was estimated at the serosurvey level using a time-varying catalytic model. Environmental, demographic and entomological predictors were used to fit and predict the FoI at municipality level from 1980 to 2010 across Colombia.

A stratified bootstrap method was used to fit the models without temporal autocorrelation at the serosurvey level. The predictive ability of each model was evaluated to select the best-fit models within urban, rural and (Amerindian) indigenous settings. Model averaging, with the 10 best-fit models identified, was used to generate predictions.

Our analysis shows a risk of overconfidence in model predictions when median estimates of FoI alone are used to fit and evaluate models, failing to account for uncertainty in FoI estimates. Our proposed methodology fully propagates uncertainty in the estimated FoI onto the generated predictions, providing realistic assessments of both central tendency and current uncertainty surrounding exposure to Chagas disease.

**Supplementary Information:**

The online version contains supplementary material available at 10.1186/s12874-021-01477-6.

## Significance statement

Estimating spatiotemporal variation in disease exposure is critical to developing cost-effective strategies to reduce disease burden. However, where there is no well-established surveillance system, it might be challenging to obtain such information. Serosurveys provide information on past exposure at a certain location but do not reflect the current situation, particularly for long-lasting diseases such as Chagas disease. The FoI provides insight into the temporal patterns of the disease and is particularly relevant for assessing spatiotemporal heterogeneities and interventions’ impacts. However, assessing incidence over countries and decades, when seroprevalence information remains limited, requires robust statistical methods. We developed a modelling framework that predicts FoI in space and time from serosurveys able to propagate uncertainties using Colombia as a case study.

## Introduction

Between 5 and 18 million persons are estimated to be currently infected by *Trypanosoma cruzi*, the protozoan parasite causing Chagas disease, and between 4200 and 33,000 per year are estimated to die in the 21 endemic countries in Latin America [1, 2]. These figures give a coarse picture of the epidemiological situation, which is problematic as reliable estimates of the spatial and temporal patterns of the disease burden are essential for governments and health organisations to assess progress towards control or elimination goals. Indeed, spatial estimates of exposure are critical to target vector control activities. Additionally, the current clinical burden depends on past exposure as people infected by *T. cruzi* may develop a chronic form of the disease, requiring long-term care. Temporal estimates of exposure to *T. cruzi* are essential to monitor diagnostic and treatment needs [3], and ultimately to coordinate intervention strategies (e.g. targeted vector control and screening interventions). Finally, temporal patterns in exposure can also be used to evaluate past control interventions and guide future planning.

Estimating the burden of Chagas disease is challenging; there are no reliable measures of incidence, for example, in Colombia, only an estimated 1.2% of the at-risk population received a screening test in 2008–2014 [4]. The low level of detection is partly linked to the unspecific nature of early symptoms and the long-lasting asymptomatic period, i.e. asymptomatic or unspecific symptoms can last for over 10 years and around 50% of those infected may never reach the chronic phase [2]. Moreover, the disease affects disproportionately poorer populations with limited access to the health system [5].

As demonstrated for other infectious diseases with a relatively low proportion of symptomatic cases, burden estimates typically rely on exposure estimates, particularly the Force-of-Infection (FoI), i.e. the per-susceptible rate of parasite acquisition [3]. Seroprevalence surveys are typically used to reconstruct past and present incidence patterns in various locations and a geostatistical model smooths the estimated FoI over space [6, 7].

Where this framework has been applied, given the complexity of the inference and relative scarcity of gound-truth data, it is common to assume that exposure has been constant over time. Although this may hold for FoI estimates for dengue [6, 8, 9], yellow fever [7], rubella [10, 11] and malaria [12], it is more challenging for Chagas disease, as its protracted nature and substantial spatial and temporal heterogeneities in the implementation of control measures lead to temporal and spatial heterogeneities in exposure.

Additionally, predicting FoI spatial patterns relies upon point estimates of FoI, with geostatistical models smoothing the central estimates [6, 7, 9], often neglecting their uncertainty. This may generate over-confidence in FoI estimates and ultimately burden. Generating FoI and disease burden estimates that robustly incorporate uncertainty is essential to inform policy-relevant questions, from affected communities to stakeholders and policy-makers [13].

Here, we propose a framework to predict spatial as well as temporal variations in FoI that fully account for uncertainties at various levels, particularly, the uncertainty in estimated FoI. The framework is applied to 76 *T. cruzi* serosurveys in Colombia to obtain estimates of exposure across Colombia from 1980 to 2014 at the municipality level. The importance of propagating uncertainty in estimated FoI and its impact on model selection and prediction was then quantified.

## Methods

### General approach

Our general aim is to predict the FoI at the municipality level across Colombia using data from 76 serosurveys (27 urban, 36 rural, 5 indigenous and 8 mixed as defined by the Colombian government) conducted between 1980 and 2014 (Supp. Fig. 1 and Supp. Fig. 2). Environmental, demographic and entomological predictors were available for each location. For each serosurvey, the full posterior distributions of the FoI were obtained using a catalytic model [3]. As a serosurvey reflects exposure since the birth of the oldest participant, estimated FoIs include past and contemporary (to the serosurvey) estimates of FoI. The potential predictors included in the models were selected based on expert knowledge and preliminary analyses (Supp. Table 1**.** presents the full list of predictors considered). Log-linear models were fitted using a combination of these predictors. Due to temporal autocorrelation, a stratified bootstrapping was applied to fit the models using single year FoI estimates (randomly chosen at each iteration). To avoid overfitting, a repeated random sub-sampling validation was applied by selecting multiple times and randomly using half of the serosurveys for either training or validation. The predictive ability of each model (i.e. central estimate across the out-of-sample sets) was then evaluated to select the best models within urban, rural and indigenous settings. Finally, model averaging, with the 10 best models identified in the 3 different settings studied, was used to produce predictions of FoI as described by [14].

Typically median, or mean, FoI estimates are used as the dependent variable [6, 7, 9]; however, ideally, the uncertainty in estimated FoIs should be accounted for when fitting the models and evaluating their predictive ability. To assess how this uncertainty impacted predictions, we compared three approaches incorporating a different level of uncertainty:

1. Central estimates of FoI are used, i.e. no uncertainty is accounted for as commonly used in the literature. The selection of the best model is based on the central trends.
2. Uncertainty in estimated FoI is used to quantify the model’s predictive ability but not for fitting. For a given model, the predictions remain the same. This approach potentially changes which models are selected as the best ones based on a more realistic measure of predictive ability.
3. Uncertainty in estimated FoI is used for both fitting and quantifying the model’s predictive ability. Models are fitted and evaluated repeatedly using samples of the FoI posterior distribution leading to changes in both the predictions for a given model and which models are selected as the best.

The uncertainty on the predictions was characterised using a coefficient of variation (CV) based on the Median Absolute Deviation (MAD) accounting for the non-normality of the FoI distribution [15]. A3, although computationally more intensive, appropriately propagates the uncertainty in FoI estimates in both the predictions and the model selection processes.

### Data input

#### Chagas disease force-of-infection

From the 112 Chagas disease serosurveys conducted in Colombia, only 76 serosurveys were selected, where the catchment area was smaller than the municipality level. Indeed, serosurveys having a catchment area at the departmental level have been excluded to be able to run analyses at the municipality level. The Force-of-Infection (FoI) is the per-susceptible rate of parasite acquisition [3] and had been estimated using Bayesian inference (to account for diagnostic uncertainty) for all those 76 age-stratified serosurveys [3]. Thus, for each serosurvey, we extracted the full posterior distribution of the estimated annual FoI from the year of birth of the oldest participant up to the year the serosurvey was conducted. The median and the 95% Bayesian Credible Intervals (CrI) were then extracted from the posterior distribution. The methodology used to calculate the FoI has been described elsewhere [3] and relies on estimating time-varying FoI based on catalytic models [16] (see SI for more details).

#### Potential explanatory variables

For each covariate, the geographical scale of interest was the municipality (ADM2) level when available or the departmental (ADM1) level, otherwise. The pool of variables tested related to both human population and environmental conditions (Supp. Table 1).

The *Trypanosoma cruzi* seroprevalence in public blood banks by year and department was provided by the Pan American Health Organization (PAHO). The presence of *Triatoma dimidiata* and *Rhodnius prolixus* at the municipality level was obtained after combining records from a national surveillance report of 2013 [17] and data from [18, 19]. We also extracted data on presence/absence of these two vector species, from which the proportion of municipalities infested for each department was calculated. Data on vector control interventions implemented in Colombia (1998–2014) were extracted from [20]. Census data were obtained from the Colombia’s Department of Statistics (DANE) website [21]. Climate variables were extracted from the Köppen-Geiger climate classification maps at a 1-km resolution [22]. Finally, the map layer used was obtained from Database of Global Administrative Areas (GADM) (https://gadm.org/ [23]).

Other covariates included the setting of the survey (urban, rural, indigenous, or mixed population (including urban, rural and unknown settings); the year when the survey was conducted; an effect for years and decades (full details in Supp. Table 1). Indigenous settings comprised those with Amerindian populations mostly following traditional lifestyles as described in [3]. Definitions for urban and rural populations followed the Colombian governement criteria [21].

### Model selection strategy

Due to temporal autocorrelation in estimated FoI, a stratified bootstrapping was applied to fit log-linear models using single year FoI estimates (randomly chosen at each iteration).

To avoid overfitting, the method of Leave-p-out cross-validation (with *p* = 50%), while ultimately ideal, was unpractical given the computational cost. Instead, we used a repeated random sub-sampling validation by selecting multiple times and randomly half of the serosurveys for either training or validation. As the number of random splits increases, the repeated random sub-sampling validation results approach the exhaustive Leave-p-out cross-validation. We used 10,000 splits to ensure convergence. The variation in the first and second 5000 out-of-sample predictive *R*^*2*^ values for the 10 best models varied by less than 1% in rural and urban settings (3% for indigenous settings).

A total of 464 models, combining 27 covariates (including some 2-ways interactions), were evaluated using the above procedure. For each model, the parameters were estimated using data from all settings (urban, rural, indigenous), but predictive performance (see below) was evaluated separately for each setting. For each setting, a model-averaging method [14] was used to account for structural uncertainties based on the 10 best models in each setting. Models’ weights based on predictive performance (see below) were used to obtain model-averaged predictions and maps.

### Modelling approaches and predictive performance

We used 2 predictive performance indicators:

- The standard predictive (out-of-sample) *R*^2^ [24] (Eq. 1),

1 $$Predictive\ {R}^2=1-\frac{\sum {\left({y}_i-\hat{y_i}\right)}^2}{\sum {\left({y}_i-\overline{y}\right)}^2}$$

- An overlap indicator estimating the percentage overlap between observed and predicted distributions (using the R-package *overlap* 1.5.4 [25].).

The predictive *R*^2^ compares the central estimate of the prediction against observations. The overlap indicator compares the full distribution of the predictions against the full distribution of the observations. Therefore, while the overlap indicator quantifies well the predicted uncertainty, the predictive *R*^2^ focuses on the central trend in observations and predictions. Model selection relied on an average of both indicators and models’ weights were adapted from [14] (Eq. 2),

2 $${w}_i=\frac{e^{\left(-\frac{1}{2}\left(\max (Ind)-{Ind}_i\right)\right)}}{\sum_{r=1}^R{e}^{\left(-\frac{1}{2}\left(\max (Ind)-{Ind}_r\right)\right)}}$$

With *R* being the total number of candidate models (here 10) and *Ind* the performance indicator.

Three modelling approaches were compared which differ in how much uncertainty in estimated FoI is accounted for while i) fitting the model and ii) assessing its predictive performance (for model selection). The 3 approaches were:

- Approach 1 (A1): only the median FoI estimates were used as the response variable, i.e. no uncertainty is used (a common approach in the literature). In this approach, as the response variable is characterised by its median, only the predictive *R*^2^ was used to select the best models. For comparison, the overlap indicator for each model was retrospectively estimated but not used.
- Approach 2 (A2): Uncertainty in estimated FoI is used to quantify the model’s predictive ability but not for fitting. In this approach, while only the median FoI is used for fitting, both the predictive *R*^2^ and the overlap indicator are used (averaged) to select the best models.
- Approach 3 (A3): Uncertainty in estimated FoI is used when both fitting and quantifying model’s predictive ability. Each model is repeatedly fitted to the posterior samples of the estimated FoI, and the predictive *R*^2^ and the overlap indicator are used (averaged) to select the best models.

### FoI prediction for the entire country

The model average built for each setting was then used to generate FoI estimates in each municipality of Colombia for the years 1980, 1990, 2000 and 2010. The median FoI and its uncertainty were extracted. The uncertainty was characterised using a standardised coefficient of variation (CV) calculated using the standardised Median Absolute Deviation (MAD) because the FoI values were not normally distributed [15].

### Comparing observations and predictions across serosurveys

For each serosurvey, we compared, across years, the median and 95%CI (Confidence Interval) of the predicted FoI against the median and 95%CrI of the originally estimated FoI [3] (i.e. the dependent variable or ‘observed’ FoI).

For each quantile of interest *q*_*x*_ (i.e., median, 2.5, and 97.5% percentiles, denoted *q*_*m*_, *q*_*l*_ and *q*_*u*_ respectively), we computed a distance between the ‘observed’ and predicted quantile (${\delta}_{q_x}$). This distance was standardised by the interval between the observed median and observed upper (or lower) 95% CrI,

3 $$\left\{\begin{array}{cc}{\delta}_{q_x}=\frac{q_x\left(\hat{y}\right)-{q}_x(y)}{q_x(y)-{q}_l(y)}& if\ {q}_x\left(\hat{y}\right)<{q}_x(y)\\ {}{\delta}_{q_x}=\frac{q_x\left(\hat{y}\right)-{q}_x(y)}{q_u(y)-{q}_x(y)}& if\ {q}_x\left(\hat{y}\right)>{q}_x(y)\end{array}\right.$$

When the predicted and ‘observed’ medians are equal, we expect ${\delta}_{q_m}=0$. If the predicted median was equal to the upper (or lower) 95%CrI of the ‘observed’ FoIs, then we would have ${\delta}_{q_m}=1\Big({\delta}_{q_m}=-(1).$

If the predicted and ‘observed’ upper (or lower) 95% CI/CrI were equal, then we expect ${\delta}_{q_u}=1$ (${\delta}_{q_u}=-1$). A value ${\delta}_{q_u}=2$ would indicate that the interval between the median and upper CI in the prediction is twice as wide as the interval between the median and upper CrI in the observations.

The change in the denominator reflects the non-symmetrical nature of the 95%CI.

As it is rescaled, this measure of bias allows an assessment of the predictive ability of our approaches across serosurveys. For each year, we estimated the median and interquartile range in the bias. This was also done by setting.

### Spatial correlation and spatial heterogeneity tests

The Spatial Correlation Diagnostic test from the PrevMap R-package (based on a permutation of locations [26]) and the Moran’s I test from spdep R-package (based on neighbourhood values [27]) were used to assess spatial auto-correlation for the best and second-best model in each setting. To analyse the spatial correlation independently from the temporal one, the tests were bootstrapped 200 times with stratification on the location (one value for each municipality by iteration).

In order to assess the spatial heterogeneity among predictions, the Moran’s I test under randomisation from spdep R-package [27] was undertaken in each setting on the predicted FoI values at the municipality level.

### Availability of data and materials

The datasets supporting the conclusions of this article are available in the repository in [28].

## Results

### Importance of accounting for the uncertainty in FoI

**Fig. 1:**
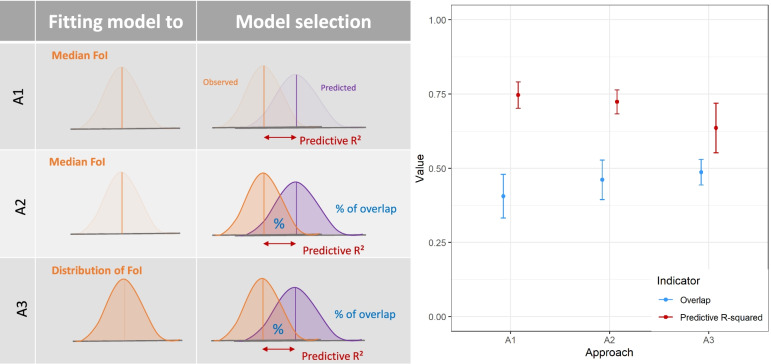
Comparison of the predictive ability of the best-fit models for the three approaches investigated. Approach 1: (A1) models fitted with median FoI estimates and selected based on predictive R^2^; Approach 2 (A2): models fitted with median FoI estimates and selected base on predictive R^2^ and overlap; Approach 3 (A3): models fitted with the full posterior distribution of FoI estimates and selected based on the predictive R^2^ and overlap. Note: The overlap obtained for A1 is presented for comparison purpose and has been calculated using the same methodology as A2 but is never taken into consideration for the model selection

**Fig. 2:**
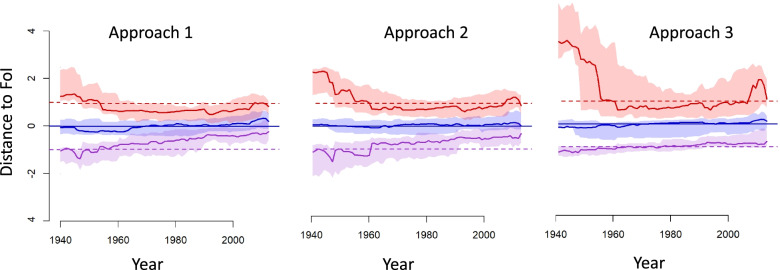
Goodness-of-fit of the model averaging of the 3 modelling approaches for all serosurveys. The solid lines and envelopes show standardised distances between observations and predictions’ median (blue), and 95%CrI (upper bound in red and lower bound in purple). A perfect fit would translate in all colored solid lines overalpping with the correspondingly-colored dotted lines. A blue solid line overlapping the blue dotted line, together with a red and purple solid lines at 2 and − 2 respectively would reflect a good central prediction with CrI in predictions twice as large as the CrI in the ‘observed’ FoI. Approach 1: models fitted with median FoI estimates and selected based on predictive R^2^; Approach 2: models fitted with median FoI estimates and selected based on predictive R^2^ and overlap; Approach 3: models fitted with the full posterior distribution of FoI estimates and selected based on the predictive R^2^ and overlap

**Fig. 3:**
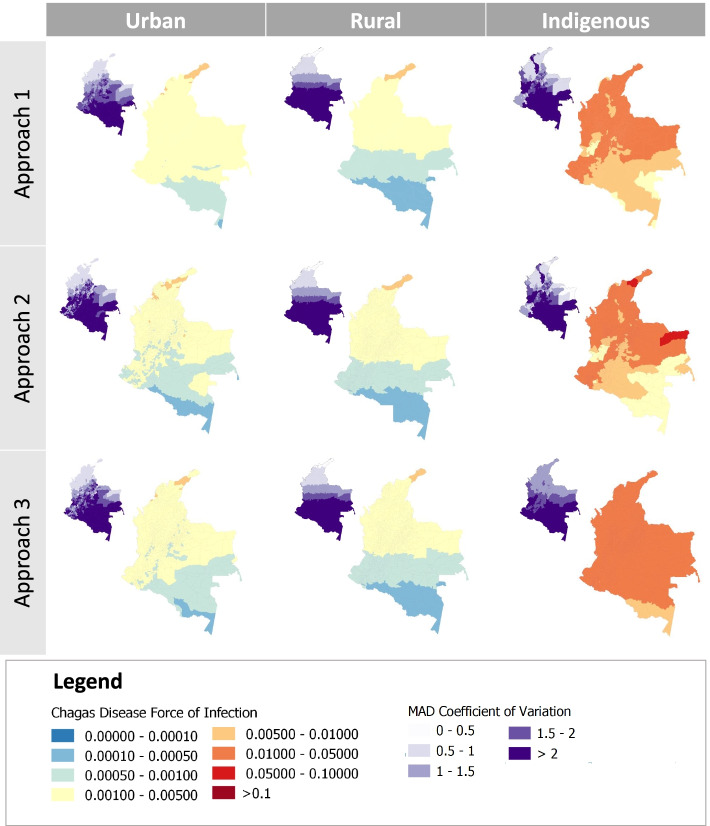
Force-of-Infection of Chagas disease in urban, rural and indigenous settings, Colombia, 1990. Main map, predictions per year and per susceptible individual; small map, Median Absolute Deviation (MAD) Coefficient of Variation (*n* = 1065 municipalities) . Rows correspond to the 3 modelling approaches. Maps show model-averaged estimates (across the 10 best setting-specific models). Approach 1: models fitted using the median FoI estimates and selected based on predictive R^2^; Approach 2: models fitted with median FoI estimates and selected based on predictive R^2^ and overlap; Approach 3: models fitted with the full posterior distribution of FoI estimates and selected based on the predictive R^2^ and overlap

When using only the central FoI estimates (A1), we obtained higher predictive R^2^ but the overlap between the predicted and estimated distribution was lower (Fig. 1 and Supp. Table 2). This is reflected in the 95% credible intervals (95%CrI) of the predicted FoI values being smaller than the 95%CrI in the original FoI estimates, indicative of substantial overconfidence in the models’ predictions (Fig. 2). This overconfidence in predictions is likely propagated to municipalities where we do not have estimates of FoI, leading to widespread overconfidence nationally (Figs. 2 and 3). This simple approach also leads to reduced heterogeneity in both space and time (Fig. 3).

In contrast, when using the full estimated distribution of FoI for both fitting and model selection (A3), we observed a lower predictive R^2^ but a greater overlap between obsevations and predictions, indicating that both the central FoI estimates and their uncertainties are well characterised (Figs. 1 and 2). This is reflected in the 95%CrI of the predicted FoIs being much closer to the 95%CrI in the originally estimated FoIs (Fig. 2 and Supp. Fig. 3). A3 did not, however, lead to higher uncertainty across municipalities, even where serological surveys have not been conducted. Using A3, we estimated that the MAD-based CV in FoI predictions was greater than 2 in 25% of municipalities (compared to 31 and 27% in 2010 for A1 and A2, respectively) (Supp. Table 3). Furthermore, the number of extreme CV values (above 5) is reduced in A3 (39, 81, 17 municipalities with CV above 5 in 1990 for A1, 2 and 3, respectively). In municipalities where serosurveys had been conducted, the median CV was higher with A3 (median CV = 1.29, 1.28 and 1.33 with approaches A1, A2 and A3, respectively), but the maximum was lower (maximum CV = 2.76 for A3, 4.06 for A1 and 4.56 for A2) (Supp. Table 4).

### Spatial and temporal predictions of FoI in Colombia

In the following, we present results from Approach 3 (unless otherwise stated); this leads to a more accurate assessment of the variations in FoI and its uncertainty. No residual spatial autocorrelation in the FoI estimates was found for any of the models as assessed by methods developed in [26, 27]; therefore, municipalities’ predictions were obtained directly from estimated models’ parameter and sets of predictors.

The FoI varied significantly by settings, with overall FoI predicted to be 9.1 and 11.8 times lower in urban and rural settings than in indigenous settings (respectively, FoI values of 2.2 × 10^− 3^, 1.7 × 10^− 3^ and 2.0 × 10^− 3^ per year and per susceptible individual).

Between 1980 and 2010, the predicted FoIs showed a decreasing trend, with relative decreases of 23, 0.07 and 7% in urban, rural and indigenous settings respectively. The decrease in predicted FoIs was statistically significant in urban and indigenous settings (Table 1 and Supp. Table 1), but not in rural settings.

**Table 1:** Predicted FoI averaged across all Colombian municipalities in 1980, 1990 and 2010, the percentage of decrease between 1980 and 2010 (trend) for each setting and the spatial clustering effect given by the Moran’s I statistic for the test under randomisation in 1980, 1990, 2000 and 2010 (*n* = 1065 municipalities)

	Predicted FoI values				Moran’s I statistic			
	1980	1990	2010	trend	1980	1990	2000	2010
	mean (sd)	mean (sd)	mean (sd)	%				
Urban	2.2 × 10^−3^ (1.1 × 10^−3^)	2.1 × 10^− 3^ (1.1 × 10^− 3^)	1.7 × 10^− 3^ (9.9 × 10^− 4^)	−23^a^	0.82	0.82	0.79	0.78
Rural	1.7 × 10^− 3^ (1.0 × 10^− 3^)	1.7 × 10^− 3^ (1.0 × 10^− 3^)	1.7 × 10^− 3^ (1.0 × 10^− 3^)	−0.07	0.93	0.93	0.93	0.93
Indigenous	2.0 × 10^− 2^ (4.5 × 10^− 3^)	2.0 × 10^− 2^ (4.5 × 10^− 3^)	1.8 × 10^− 2^ (4.4 × 10^− 3^)	−7^a^	0.91	0.91	0.90	0.90

^a^Statistically significant at a 5% significance level according to Student’s *t* test comparing FoI values between 1980 and 2010

**Fig. 4:**
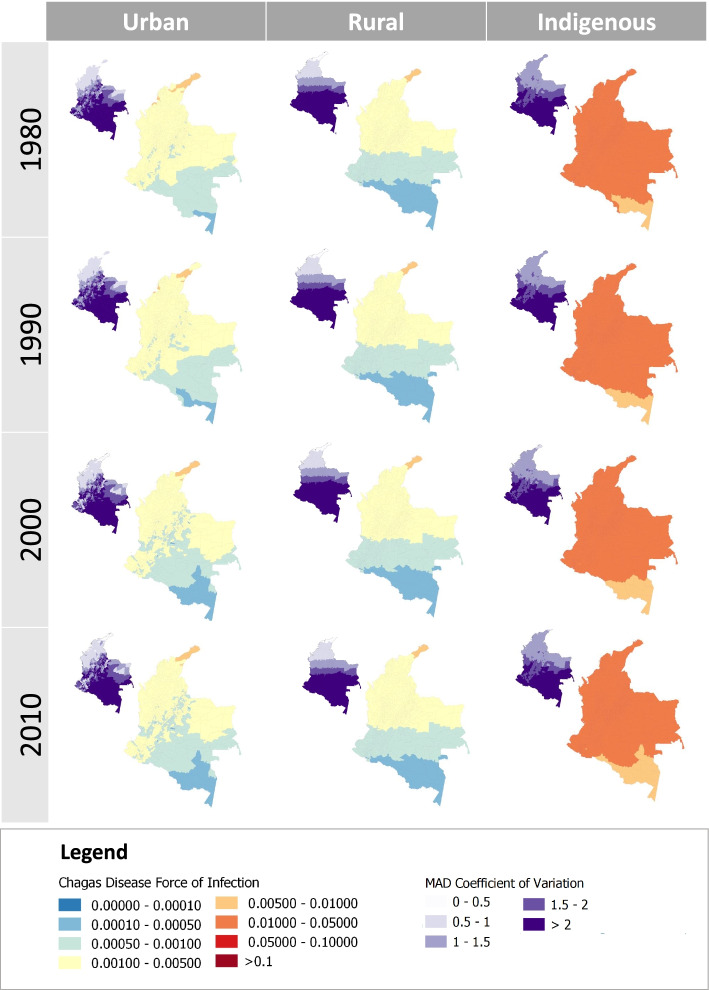
Spatiotemporal trends in Chagas disease Force-of-Infection, Colombia, 1980–2010. Main maps, predictions per year using approach 3 and model averaging; small maps, MAD Coefficient of Variation (*n* = 1065 municipalities)

Spatially, rural FoIs showed a clear north–south gradient, with estimated FoI values per year reaching 0.05–0.01 in the north compared to 0.0001 in the most southern municipalities (Fig. 4). In all settings, the uncertainty estimated was higher in the most southern municipalities. In 1990, the Moran’s I test under randomisation shows that there was spatial clustering in the predicted FoIs. The heterogeneity in predicted FoI was higher in urban settings (Moran’s I statistic value of 0.82) than in rural setting (Moran’s I statistic value of 0.93). In addition, the clustering effect seemed to decrease over time in urban settings, but not in rural ones (Moran’s I statistic in urban settings in 1980 is 0.82 while it is 0.78 in 2010).

### Main predictors of Trypanosoma cruzi exposure

**Fig. 5:**
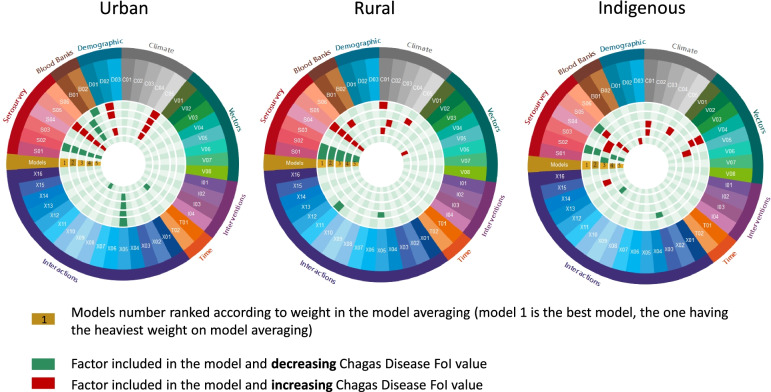
Predictors included in the model averaging of the FoI of Chagas disease in Colombia. Models fitted with the full posterior distribution of FoI estimates and selected based on predictive R^2^ and overlap. For the full set of predictors see Supp. Table 1

Model complexity was similar across settings, with the number of predictors included in the 10 best-fit models varying from 10 to 14 in urban settings, 7–13 in rural settings and 6–12 in indigenous settings (Fig. 5).

In urban and rural areas, the predictors selected in each of the 5 best-fit models were consistent, with small changes from one model to another; while in indigenous settings, models were more distinct.

The urban-setting models always included the setting of the survey (urban, rural and indigenous) (S01), as well as its latitude (S05). Seroprevalence in blood banks and climate variables were included in 4 out of the 5 models. The level of poverty (D02) was selected and positively correlated with FoI in 3 models out of the 5 models. The interaction between the prevalence in blood banks and tropical climate (X05) was selected in 4 of the models. The year and the interaction between the amount of vector control interventions and the proportion of municipalities infested by *Triatoma dimidiata* were both included in one of the models.

The rural-setting models always included the year when the serosurvey was conducted (S01), as well as the setting (urban, rural or indigenous) (S02) and its latitude (S05). Four out of the 5 models included a climate variable. Blood bank and vector variables were only included once. Demographic, vector interventions and time variables were never selected in rural models, not even as interaction terms. Only two interactions were included; the interaction between prevalence in blood banks and tropical climate (X05), and the proportion of municipalities infested by *Rhodnius prolixus* and longitude (X11).

The indigenous-setting models were far more varied. The year when the serosurveys were conducted (S01) was included in one model. The setting was always included (S02 and S03/S04) but one of the models used the indigenous setting (S03) and the urban setting (S04) against the others as risk factors. The effect of latitude (S05) was not as clear as for urban and rural settings. Poverty (D02) was the only demographic variable included directly, but the population density was included in interaction terms with the prevalence in blood banks (X03). Vector variables played an important role in three models. These predictors were also included as interaction terms in X11 (the proportion of municipalities infested by *R. prolixus* and longitude) and X14 (*T. dimidiata* density and vector-control interventions).

While all the best-fit models selected for prediction in rural settings included a predictor specifying the year when the serosurvey was conducted (S01, Fig. 5), this variable was not included in any of the best models for predictions in urban settings and was included in only one of the models for indigenous settings. Consistently, for a given year and municipality, the predicted FoI values from older serosurveys were higher than those of more contemporary serosurveys (Supp. Fig. 4). The inclusion of the year of the survey as predictor for rural settings highlights potentially a bias in sampling, with older serosurveys being less representative and biased toward municipalities with higher FoI (Supp. Table 6 and Supp. Fig. 4).

## Discussion

We predicted spatial and temporal variations in FoI across Colombia based on estimated FoI from 76 serosurveys conducted between 1980 and 2014. Our analysis highlights the importance of accounting for the uncertain nature of the estimated FoI by demonstrating a substantial risk of overconfidence when using median estimates of FoI to fit and evaluate models, as typically done in the literature [6, 7, 9]. We propose a novel methodology to fully propagate uncertainty from the estimated FoI onto the predicted one, giving a realistic assessment of both the central tendency and uncertainty surrounding past and current exposure to Chagas disease across Colombia.

Accounting for and communicating uncertainty in FoI estimates is critical to better inform public health and clinician stakeholders [13]. It allows a better assessment of where information is missing, rather than giving a false sense of certainty. Our framework offers the opportunity to prioritise areas where serosurveys would be needed. In addition, where uncertainty is low, the models identified areas where we can be confident that populations have experienced, or are experiencing, high exposure to *T. cruzi*, which is critical to better inform focused interventions for patient diagnosis and care.

The performances of the models obtained were good, with performance indicators measuring the predictive ability of both central trends and uncertainty, estimated to vary between 0.46 and 0.67 for the five best-fit models (Supp. Table 2). When predicting FoI in new areas (where serosurveys have not yet been conducted), the uncertainty, characterised by the CV, can become much larger, while the median remains consistent across settings (in 1990, urban: median CV = 1.48, range CV = 0.32–8.19; rural: median CV = 1.50, range CV = 0.24–11.00; indigenous: median CV = 1.50, range CV = 1.07–3.52). In contrast, Garske et al. obtained FoI predictions of yellow fever with a CV ranging from 0 to 3 using central estimates of the FoI to fit their model. Using the same methodology (i.e. Approach 1), our results showed similar median uncertainty (urban: median CV = 1.48, range = 0.34–6.05; rural: median CV = 1.51, range = 0.23–11.98). To some extent, the relatively smaller uncertainty obtained in the context of yellow fever by Garske et al. might also be explained by their assumption of a constant FoI over time, rather than the time-varying FoI we used in this work for Chagas disease. Given the demographic and public health changes that have occurred in Colombia over the past decades (considerable rural-to-urban migration, housing improvements, scaled-up vector control, more efficient diagnostic protocols), we believe that accounting for temporal variations in Chagas disease FoI is critical for our analysis, even at the ‘cost’ of increased uncertainty.

At first glance, our analysis highlights some unexpected results. The effect of time was relatively weak, i.e. with FoI not showing a significant decrease in rural settings; as was the effect of rural vs. urban settings. Such results contrast with previous evidence, which showed a strong temporal trend [3, 29–31], and increased exposure in rural settings where vectorial transmission is much more prevalent [3, 30, 31]. In terms of temporal trends, our final models always include time-varying variables, such as poverty levels and vector density, which have decreased over time, due to intervention implementation and general improvement of living conditions in the country. However, we showed that the year when the serosurvey was conducted impact the estimated FoI, with older serosurveys biased toward high-risk areas (Supp. Fig. 4). Regarding the lack of substantial differences in the level of exposure between rural and urban settings, the great population migration trends observed across the country are likely blurring this effect. Considerable rural to urban migration has taken place in Colombia, with one-third of the rural population aged below 40 in 1951 having migrated to urban settings by 1964, mostly to find better employment opportunities [32]. More recently, it has been estimated that more than 3.5 million people had migrated to urban centres to escape violence in rural areas [33]. Having lived for an extended period of time in rural settings, these migrants may well have been exposed to *T. cruzi* in rural areas but now account for the estimated FoI in urban settings. Unfortunately, the participants’ migration history was not recorded (or available) in the serosurveys used. Similar dynamics of migrations have been shown to explain a substantial burden of Chagas disease in both endemic (e.g. in Arequipa, Peru [34]) and non-endemic settings [35].

Another spatial challenges is the scale at which the analyses have been conducted. Indeed, we demonstrate small-scale spatial heterogeneity in Chagas disease exposure between the municipalities within a department. And, while our approach was designed to be consertvative by excluding serosurveys providing information only at the departmental level, we acknowledge that further small-scale heterogeneity may exist, i.e. difference could occur between villages of the same municipality. However, the municipal level is the operational level in the control of Chagas disease and is, therefore, the most useful level to characterize exposure in a way that actionable information can be extracted. Also,we found that most of the important variables for predictions were available at the municipality level (poverty indicator, vector density), but not disaggregated further. Thus, even if a small-scale analysis could provide some insights, technically and operationally, the municipal level remain the most relevant one.

While serosurveys provide invaluable information on exposure, our analysis highlights the importance of appropriate sampling strategies. Sampling decisions taken to collect the data have a clear impact on our ability to provide representative predictions over large spatial and temporal scales. One issue linked to sample representativeness is the location of the serosurveys. Indeed, the likely past focus on estimating exposure in high-risk populations may have created a selection bias that cannot be easily handled when modelling the data. In Colombia, this seemed especially true in rural settings (Supp. Fig. 4). This bias likely explains much of the temporal trends that have been reported in previous studies (e.g. [3]). This highlights the problem of relying on surveys that were not designed to provide a representative sample but rather organised to confirm and quantify incidence in high-risk areas. Extrapolation to areas where no serosurveys have been conducted is then made more uncertain and need to be interpreted accordingly. Another issue linked to sample representativeness is the targeted age groups of the surveys. In 2012, the World Health Organization set elimination of (intradomicilary) Chagas disease transmission as a goal in its first neglected tropical disease roadmap; one of the indicators used to monitor progress towards this goal was the seroprevalence among under-five children, aiming to measure active transmission as opposed to past exposure [3, 36]. Unfortunately, such (narrow age-range) sampling scheme hampers obtaining valuable information about past exposure, which for a chronic illness, such as Chagas disease, is crucial to target diagnosis and treatment. We argue that organising representative serosurveys and covering a broader age range is essential to obtain a reliable picture of the epidemiological situation and the impact of control interventions in endemic countries, particularly for infectious diseases that use serosurveys for the purposes of surveillance.

## Supplementary Information

**Additional file 1.**

## Data Availability

The datasets generated and/or analysed during the current study are available in the GitHub repository for Chagas disease FoI with Linear Models, https://github.com/jledien/Chagas-disease-FoI-with-Linear-Models.git [28].

## Abbreviations

CrI: Bayesian Credible Intervals
CV: Coefficient of variation
DANE: Colombia’s Department of Statistics
CI: Confidence Interval
GADM: Database of Global Administrative Areas
FoI: Force-of-Infection
MAD: Median Absolute Deviation
PAHO: Pan American Health Organization
