# Supplementary material for: Spatiotemporal variations in exposure: Chagas disease in Colombia as a case study
Source: BMC Med Res Methodol. 2022 Jan 13;22:13. doi: 10.1186/s12874-021-01477-6 (PMC8759231; doi:10.1186/s12874-021-01477-6)
Supplement: Supplementary file 1 — Additional file 1. [file 12874_2021_1477_MOESM1_ESM.docx]

**Supplementary information**

Spatiotemporal variations in exposure: Chagas disease in Colombia as a case study

Julia Ledien^1^, Zulma M. Cucunubá^2,3^, Gabriel Parra-Henao^4,5^, Eliana Rodríguez-Monguí^6^, Andrew P. Dobson^7^, María-Gloria Basáñez^2^, Pierre Nouvellet^1^

Table of contents

**Supplementary tables**

[Supp. Table 1:Variables tested as factors in the geospatial analyses of Chagas disease in Colombia 3](#_Toc76979438)

[Supp. Table 2: Final performances of the 5 best models for each of the three settings investigated for the 3 different approaches 7](#_Toc76979439)

[Supp. Table 3: Number of municipalities where the MAD Coefficient of Variation of the predictions of the model averaging is above 2 for each of the 3 approaches and for predictions in 1980 and 2010. 7](#_Toc76979440)

[Supp. Table 4: Median values of the MAD Coefficient of Variation (CV) of the predictions of the model averaging in areas where serosurveys have been conducted (in catchment area) and where no data were available (out of catchment area) and number of municipalities where the CV is greater than 5 8](#_Toc76979441)

[Supp. Table 5: Predicted FoI averaged across all Colombian municipalities and among municipalities where serosurveys have been conducted and used in the analyses in 1980, 1990 and 2010, the percentage of decrease between 1980 and 2010 (trend) 8](#_Toc76979442)

[Supp. Table 6: Comparison of the observed FoI of Chagas Disease for serosurveys organised before and after 2005 in urban, rural and mixed settings, Colombia, 1998-2014. 9](#_Toc76979443)

**Supplementary** **figures**

[Supp. Figure 1: Locations and sample sizes of Chagas disease serosurveys organised in Colombia, 1980‒2014 and boxplot of the Force-of-Infection values (per year and per susceptible) by settings 11](#_Toc77253568)

[Supp. Figure 2: Locations and sample sizes of Chagas disease serosurveys conducted in Colombia at ADM2 level, 1980‒2014 12](#_Toc77253569)

[Supp. Figure 3: Goodness of fit of the model averaging of the 3 modelling approaches for each setting (urban, rural and indigenous). The lines and envelopes are the distance between observations and predictions’ median (blue), and 95%CI (upper bound in red and lower bound in purple); Approach 1: models fitted with median FoI estimates and selected based on Predictive R^2^; Approach 2: models fitted with median FoI estimates and selected based on Predictive R^2^ and overlap; Approach 3: models fitted with the full posterior distribution of FoI estimates and selected based on the Predictive R^2^ and overlap. 13](#_Toc77253570)

[Supp. Figure 4 :Averaged observed FoI values by year for serosurveys conducted before 2010 in blue (n=39) and after or in 2010 in pink (n=40) for a) all settings (urban, rural, indigenous and mixed), b) rural only and c) urban only. 14](#_Toc77253571)

**Supplementary methods**

Models used to estimate the Force-of-Infection: Extracted from Z. M. Cucunubá, “Modelling the epidemiology and healthcare burden of Chagas disease in Colombia,” Imperial College of London. (2017) …………………………………………………………………………………………………………………………………………………14

**Supplementary Tables**

Supp. Table 1:Variables tested as factors in the geospatial analyses of Chagas disease in Colombia

| Code | Name | Description | Spatial scale | Data Source |
| --- | --- | --- | --- | --- |
|  | **Serosurvey characteristics:** |  |  |  |
| S01 | Year of the survey | Year when the serosurvey was conducted | ‒ | (1) |
| S02 | Setting | Setting of the serosurvey: urban, rural, indigenous or mixed | ‒ | (1) |
| S03 | Urban setting | Yes/No | ‒ | (1) |
| S04 | Indigenous setting | Yes/No | ‒ | (1) |
| S05 | Latitude | latitude of the centroid of the catchment area of the serosurvey | ‒ | (1) |
| S06 | Longitude | longitude of the catchment area of the serosurvey | ‒ | (1) |
|  | **Blood banks data:** |  |  |  |
| B01 | Seroprevalence | Number of blood units positive for *T. cruzi* divided by the number of blood units tested. Data aggregated for 1993– 2010 by department | Department | PAHO |
| B02 | Proportion of blood units screened | Number of blood units tested for *T cruzi* divided by the number of blood units received. Data aggregated for 1993– 2010 by department | Department | PAHO |
|  | **Demography:** |  |  |  |
| D01 | Population density | Estimates of the annual population size at municipality level from the government divided by the surface of the municipality in km^2^ extracted from GDAM shapefiles | Municipality | (2) |
| D02 | Poverty | Proportion of households with deficit from 1993 census for 1950‒1999 and from 2005 census for 2000‒2014 | Municipality | (2) |
| D03 | Rural Indigenous Population size | Population size of the indigenous community living in rural areas from 2005 census | Department | (2) |
|  | **Climate:** |  |  |  |
|  | *Continuous* |  |  |  |
| C01 | Polar climate frequency | Number of pixels defined as polar climate divided by the total number of pixels in the municipality | Municipality | (3) |
| C02 | Tropical climate frequency | Number of pixels defined as tropical climate divided by the total number of pixels in the municipality | Municipality | (3) |
| C03 | Temperate climate frequency | Number of pixels defined as temperate climate divided by the total number of pixels in the municipality | Municipality | (3) |
| C04 | Arid climate frequency | Number of pixels defined as arid climate divided by the total number of pixels in the municipality | Municipality | (3) |
|  | *Categorical* |  |  |  |
| C05 | Tropical climate categorized | Tropical climate frequency categorized as follows: low (<10%), medium (10%‒60%), large (60%‒90%) and extra‒large (>90%) | Municipality | (3) |
|  | **Entomological data:** |  |  |  |
|  | *At departmental level* |  |  |  |
| V01 | *R.* *prolixus* geographical extent* | Number of municipalities where *R*. *prolixus* is present divided by the number of municipalities in the department. Combined data from National report of 2013 and more recent data from Parra-Henao *et al*. | Department | (4–6) |
| V02 | *T. dimidiata* geographical extent* | Number of municipalities where *T.* *dimidiata* is present divided by the number of municipalities in the department. Combined data from National report of 2013 and more recent data from Parra-Henao *et al.* | Department | (4–6) |
| V03 | *R.* *prolixus* presence | Presence of *R.* *prolixus* in the department (yes/no). Combined data from National report of 2013 and more recent data from Parra-Henao *et al*. | Department | (4–6) |
| V04 | *T*. *dimidiata* presence | Presence of *T.* *dimidiata* in the department (yes/no). Combined data from National report of 2013 and more recent data from Parra-Henao *et al.* | Department | (4–6) |
|  | *At municipality level* |  |  |  |
| V05 | *R.* *prolixus* density | Number of *R.* *prolixus* specimens found divided by the number of households in the municipality. Data extracted from the National report of 2013 | Municipality | (4) |
| V06 | *T.* *dimidiata* density | Number of *T.* *dimidiata* specimens found divided by the number of households in the municipality. Data extracted from the National report of 2013 | Municipality | (4) |
| V07 | *R. prolixus* presence | Presence of *R. prolixus* in the municipality (yes/no). Combined data from National report of 2013 and more recent data from Parra-Henao *et al*. | Municipality | (4–6) |
| V08 | *T. dimidiata* presence | Presence of *T. dimidiata* in the municipality (yes/no). Combined data from National report of 2013 and more recent data from Parra-Henao *et al*. | Municipality | (4–6) |
|  | **Interventions:** |  |  |  |
|  | *At Municipality level* |  |  |  |
| I01 | Intervention intensity | Number of municipalities where interventions were organized divided by the number of municipalities in the department for the following time periods: before 1996, 1996‒2000, 2001‒2010 and 2011‒2014. | Department | (7) |
| I02 | Intervention category | Municipality-level intervention intensity categorized as follows: no intervention (0%), low (0%‒25%), medium (25%‒50%), high (50%‒75%), very high (>75%) | Department | (7) |
|  | *At Household level* |  |  |  |
| I03 | Household intervention | Number of households having received interventions divided by the total number of households in the department for the following time periods: before 1996, 1996‒2000, 2001‒2010 and 2011‒2014. | Department | (7) |
| I04 | Household intervention category | Household-level intervention intensity categorized as follows: no intervention (0%), medium (0%‒10%), high (>10%) | Department | (7) |
|  | **Time:** |  |  |  |
| T01 | Year | Year of the FoI value | ‒ | ‒ |
| T02 | Decade | Decade of the FoI value defined as follows: 1[1900‒1909], 2[1910‒1919], 3[1920‒1929], 4[1930‒1939], 5[1940‒1949], 6[1950‒1959], 7[1960‒1969], 8[1970‒1979], 9[1980‒1989], 10[1990‒1999], 11[2000‒2009], 12[2010‒2014] | ‒ | ‒ |
|  | **Interactions:** |  | | |
| X01 | D01 x D02 |  | | |
| X02 | B01 x T02 |  | | |
| X03 | B01 x D01 |  | | |
| X04 | B02 x C05 |  | | |
| X05 | B01 x C05 |  | | |
| X06 | B02 x V01 |  | | |
| X07 | B01 x V05 |  | | |
| X08 | V08 x I04 |  | | |
| X09 | V02 x I01 |  | | |
| X10 | V02 x I04 |  | | |
| X11 | V01 x S06 |  | | |
| X12 | V05 x S06 |  | | |
| X13 | V07 x S06 |  | | |
| X14 | V06 x I04 |  | | |
| X15 | C04 x I02 |  | | |
| X16 | C05 x I02 |  | | |

Note: Predictors at the departmental level are included in the model at the departmental level, i.e. each municipality receive the departmental value.

Supp. Table 2: Final performances of the 5 best models for each of the three settings investigated for the 3 different approaches

|  | Approach 1 | | |  | Approach 2 | | |  | Approach 3 | | |
| --- | --- | --- | --- | --- | --- | --- | --- | --- | --- | --- | --- |
|  | Urban | Rural | Ind. |  | Urban | Rural | Ind. |  | Urban | Rural | Ind. |
| 1^st^ models |  |  |  |  |  |  |  |  |  |  |  |
| Indicator | 0.827 | 0.781 | 0.672 |  | 0.638 | 0.541 | 0.637 |  | 0.670 | 0.5475 | 0.526 |
| Predictive *R*² | 0.827 | 0.781 | 0.672 |  | 0.718 | 0.768 | 0.672 |  | 0.776 | 0.708 | 0.505 |
| Overlap | - | - | - |  | 0.558 | 0.314 | 0.601 |  | 0.563 | 0.387 | 0.546 |
| 2^nd^ models |  |  |  |  |  |  |  |  |  |  |  |
| Indicator | 0.821 | 0.781 | 0.649 |  | 0.631 | 0.536 | 0.619 |  | 0.667 | 0.5430 | 0.524 |
| Predictive *R*² | 0.821 | 0.781 | 0.649 |  | 0.827 | 0.685 | 0.642 |  | 0.777 | 0.599 | 0.500 |
| Overlap | - | - | - |  | 0.435 | 0.387 | 0.595 |  | 0.556 | 0.487 | 0.548 |
| 3^rd^ models |  |  |  |  |  |  |  |  |  |  |  |
| Indicator | 0.821 | 0.778 | 0.642 |  | 0.630 | 0.535 | 0.606 |  | 0.654 | 0.5425 | 0.464 |
| Predictive *R*² | 0.821 | 0.778 | 0.642 |  | 0.821 | 0.771 | 0.617 |  | 0.771 | 0.721 | 0.402 |
| Overlap | - | - | - |  | 0.439 | 0.298 | 0.595 |  | 0.537 | 0.364 | 0.526 |
| 4^th^ models |  |  |  |  |  |  |  |  |  |  |  |
| Indicator | 0.819 | 0.776 | 0.623 |  | 0.626 | 0.534 | 0.599 |  | 0.652 | 0.5425 | 0.460 |
| Predictive *R*² | 0.819 | 0.776 | 0.623 |  | 0.802 | 0.771 | 0.615 |  | 0.741 | 0.718 | 0.404 |
| Overlap | - | - | - |  | 0.449 | 0.297 | 0.582 |  | 0.562 | 0.367 | 0.515 |
| 5^th^ models |  |  |  |  |  |  |  |  |  |  |  |
| Indicator | 0.818 | 0.771 | 0.617 |  | 0.625 | 0.533 | 0.597 |  | 0.628 | 0.5415 | 0.456 |
| Predictive *R*² | 0.818 | 0.771 | 0.617 |  | 0.719 | 0.776 | 0.649 |  | 0.781 | 0.728 | 0.402 |
| Overlap | - | - | - |  | 0.531 | 0.289 | 0.545 |  | 0.474 | 0.355 | 0.510 |

Ind. = Indigenous.

Supp. Table 3: Number of municipalities where the MAD Coefficient of Variation of the predictions of the model averaging is above 2 for each of the 3 approaches and for predictions in 1980 and 2010.

|  | Approach 1 | | | |  | Approach 2 | | | |  | Approach 3 | | | |
| --- | --- | --- | --- | --- | --- | --- | --- | --- | --- | --- | --- | --- | --- | --- |
|  | 1980 | | 2010 | |  | 1980 | | 2010 | |  | 1980 | | 2010 | |
|  | n | % | n | % |  | n | % | n | % |  | n | % | n | % |
| Urban | 163 | 15.31 | 243 | 2282 |  | 359 | 33.71 | 354 | 33.24 |  | 286 | 26.85 | 299 | 28.08 |
| Rural | 285 | 26.76 | 284 | 26.67 |  | 271 | 25.45 | 275 | 25.82 |  | 266 | 24.98 | 265 | 24.88 |
| Indigenous | 341 | 3202 | 334 | 31.36 |  | 337 | 31.64 | 348 | 32.68 |  | 239 | 22.44 | 236 | 22.16 |
|  |  |  |  |  |  |  |  |  |  |  |  |  |  |  |
| Total | 789 | 24.69 | 861 | 26.95 |  | 967 | 30.27 | 977 | 30.58 |  | 791 | 27.76 | 800 | 25.04 |

Supp. Table 4: Median values of the MAD Coefficient of Variation (CV) of the predictions of the model averaging in areas where serosurveys have been conducted (in catchment area) and where no data were available (out of catchment area) and number of municipalities where the CV is greater than 5

|  | Median CV values (range) | |  | Number of municipalities with CV> 5 | | |
| --- | --- | --- | --- | --- | --- | --- |
|  | in catchment area | out catchment area |  | urban | rural | all |
| A1 | 1.29 (0.43-4.06) | 1.49 (0.23-11.98) |  | 2 | 14 | 39 |
| A2 | 1.28 (0.44-4.56) | 1.49 (0.16-12.12) |  | 9 | 13 | 81 |
| A3 | 1.33 (0.44-2.76) | 1.49 (0.24-11.00) |  | 6 | 11 | 17 |

Supp. Table 5: Predicted FoI averaged across all Colombian municipalities and among municipalities where serosurveys have been conducted and used in the analyses in 1980, 1990 and 2010, the percentage of decrease between 1980 and 2010 (trend)

|  | All Municipalities | | | |  | Municipalities in catchment area | | | |
| --- | --- | --- | --- | --- | --- | --- | --- | --- | --- |
|  | 1980 | 1990 | 2010 | trend |  | 1980 | 1990 | 2010 | trend |
|  | mean  (sd) | mean  (sd) | mean  (sd) | % |  | mean (sd) | mean  (sd) | mean (sd) | % |
| Urban | 2.2 x 10^-3^  (1.1 x 10^-3^) | 2.1 x 10^-3^  (1.1 x 10^-3^) | 1.7 x 10^-3^  (9.9 x 10^-4^) | -23* |  | 2.2 x 10^-3^  (9.6 x 10^-4^) | 2.1 x 10^-3^  (9.1 x 10^-4^) | 1.6 x 10^-3^  (8.7 x 10^-4^) | -25* |
| Rural | 1.7 x 10^-3^ (1.0 x 10^-3^) | 1.7 x 10^-3^  (1.0 x 10^-3^) | 1.7 x 10^-3^  (1.0 x 10^-3^) | -0.07 |  | 1.7 x 10^-3^  (6.3 x 10^-4^) | 1.7 x 10^-3^  (6.3 x 10^-4^) | 1.7 x 10^-3^  (6.3 x 10^-4^) | -0.10 |
| Indigenous | 2.0 x 10^-2^  (4.5 x 10^-3^) | 2.0 x 10^-2^ (4.5 x 10^-3^) | 1.8 x 10^-2^ (4.4 x 10^-3^) | -7* |  | 2.3 x 10^-2^ (2.9 x 10^-3^) | 2.3 x 10^-2^ (2.9 x 10^-3^) | 2.1 x 10^-2^ (3.0 x 10^-3^) | -9* |

*Statistically significant at a 5% significance level according to Student’s *t* test comparing FoI values between 1980 and 2010

NB. The average FoI estimates are significantly higher before 2010 than after 2010 in all settings (Supp. Table 6) meaning that the year when the serosurvey was organised impacted all the settings with the greater impact in the rural settings.

Supp. Table 6: Comparison of the observed FoI of Chagas Disease for serosurveys organised before and after 2005 in urban, rural, indigenous and mixed settings, Colombia, 1998-2014.

|  | 2010 | | | | | | | | | | | | |
| --- | --- | --- | --- | --- | --- | --- | --- | --- | --- | --- | --- | --- | --- |
|  | Before | | | | |  | After | | | | |  | t-test |
|  | n | min | med | mean | max |  | n | min | med | mean | max |  | p_value |
| Urban | 368 | 0.0005 | 0.0029 | 0.0031 | 0.0089 |  | 421 | 0.0003 | 0.0012 | 0.0017 | 0.0055 |  | << 0.001 |
| Rural | 433 | 0.0005 | 0.0035 | 0.0053 | 0.0166 |  | 566 | 0.0002 | 0.0008 | 0.0013 | 0.0064 |  | << 0.001 |
| Indigenous | 230 | 0.0196 | 0.0279 | 0.0342 | 0.0772 |  | 18 | 0.0196 | 0.0201 | 0  0203 | 0.0215 |  | << 0.001 |
| Mixed | 99 | 0.0019 | 0.0061 | 0.0051 | 0.0075 |  | 218 | 0.0001 | 0.0006 | 0.0025 | 0.0081 |  | << 0.001 |

**Supplementary Figures**

In Colombia, 109 serosurveys were conducted after 1980 and they only provide information on the catchment area, either at Municipality or Departmental levels. The presence of domiciliated vectors, which transmit most of the infection, can vary greatly from one area to another. Even at the village level, vector infestation strongly depends on the materials used to build houses, as well as on knowledge of the risk and vector control activities. Therefore, in this analyses, we only used the serosurveys with information on the location at municipality level. Being able to use all the data available as well as using a smaller geographical scale would provide a more coherent model. This is not possible within the linear framework but some more advanced methods, such as machine learning, could handle this issue.

From the 76 serosurveys used in the analyses, 27 were conducted in urban settings, 36 in rural settings, 5 in indigenous settings and 8 were mixed (including urban, rural, and unknown settings).


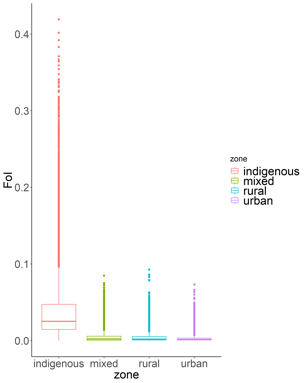

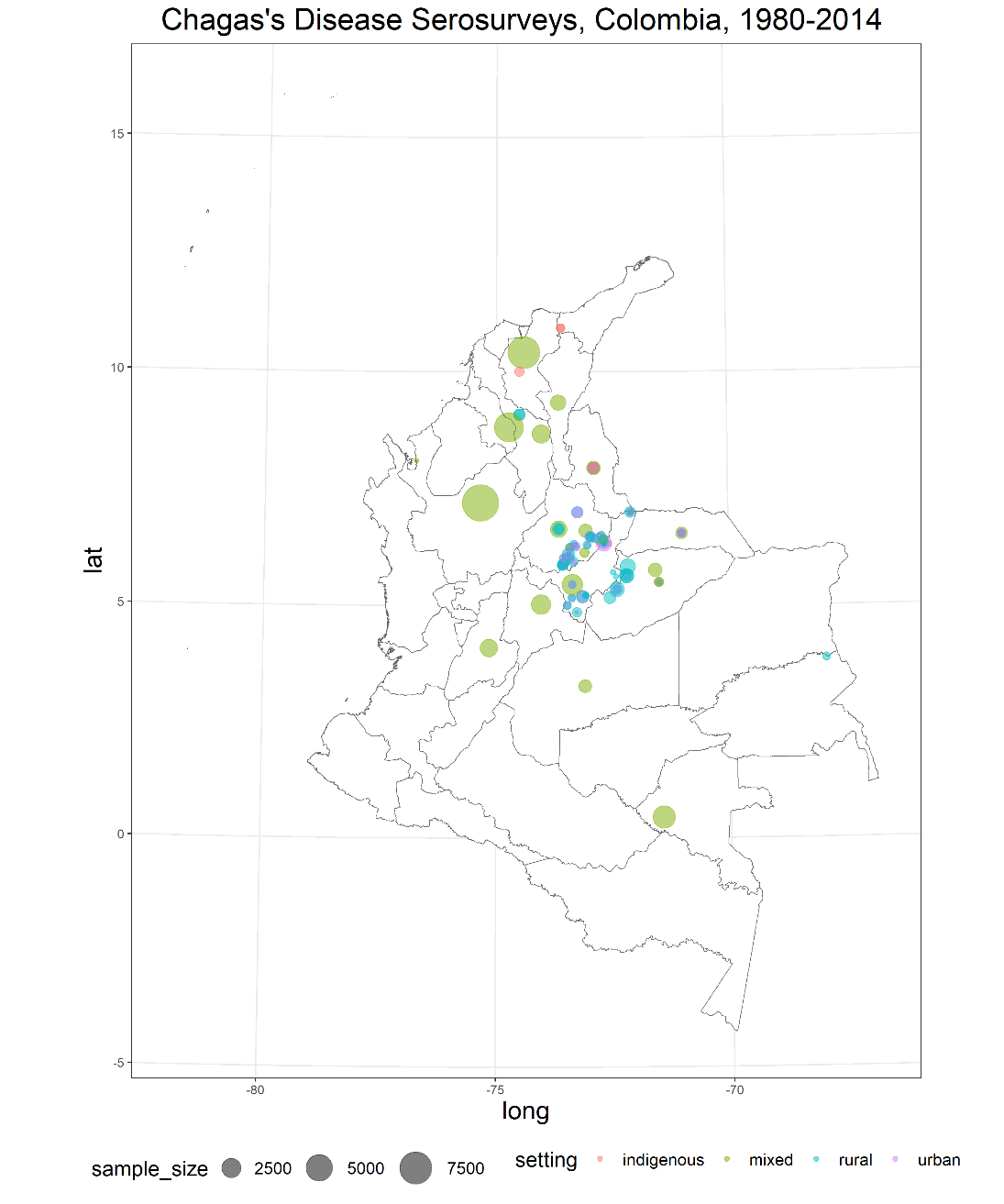


Supp. Figure 1: Locations and sample sizes of Chagas disease serosurveys organised in Colombia, 1980‒2014 and boxplot of the Force-of-Infection values (per year and per susceptible) by settings


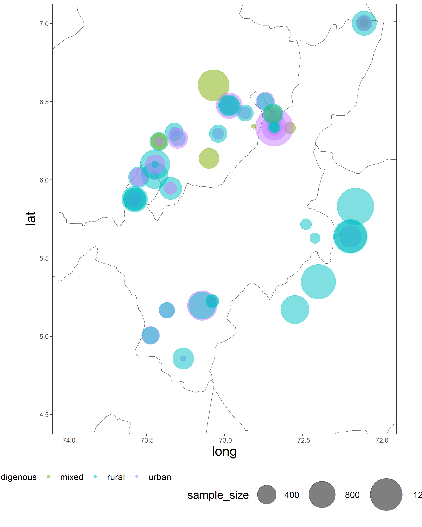

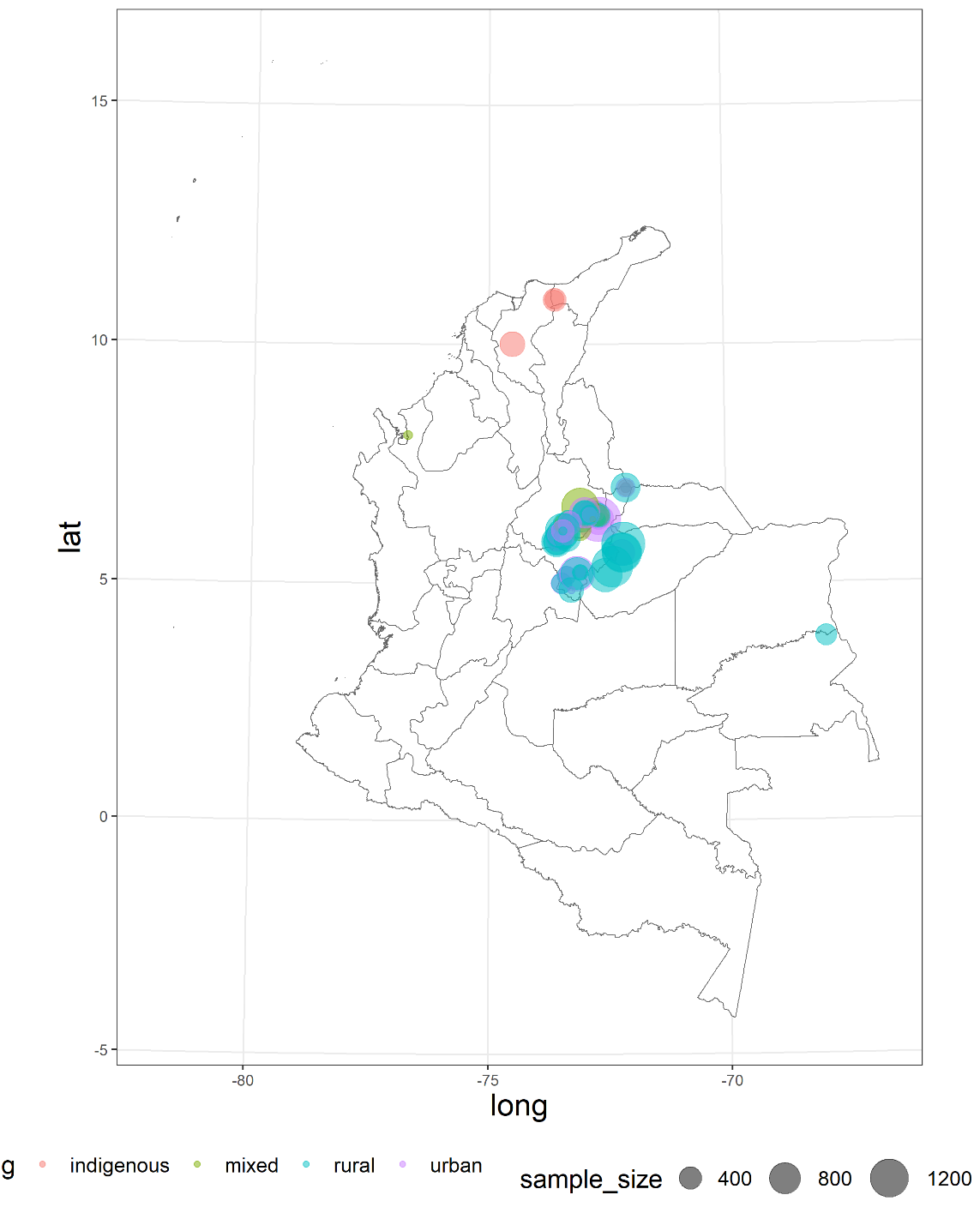


Supp. Figure 2: Locations and sample sizes of Chagas disease serosurveys conducted in Colombia at ADM2 level, 1980‒2014


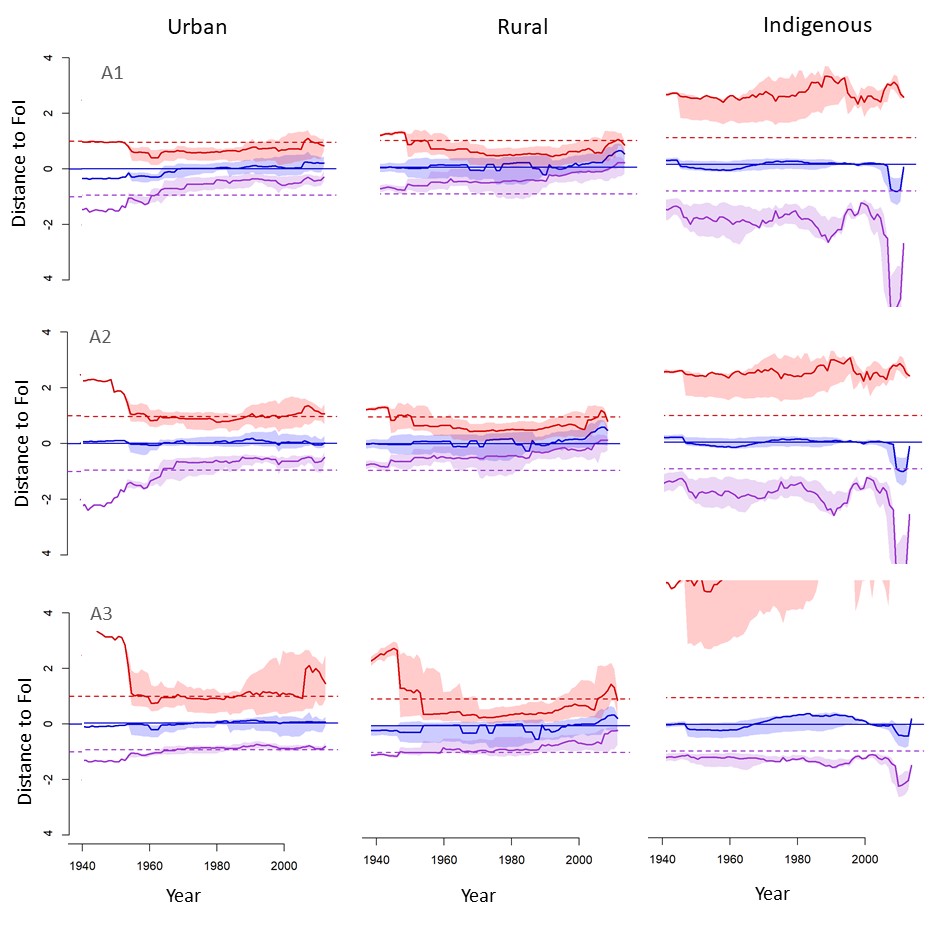


Supp. Figure 3: Goodness of fit of the model averaging of the 3 modelling approaches for each setting (urban, rural and indigenous). The lines and envelopes are the distance between observations and predictions’ median (blue), and 95%CI (upper bound in red and lower bound in purple); Approach 1: models fitted with median FoI estimates and selected based on Predictive R^2^; Approach 2: models fitted with median FoI estimates and selected based on Predictive R^2^ and overlap; Approach 3: models fitted with the full posterior distribution of FoI estimates and selected based on the Predictive R^2^ and overlap.


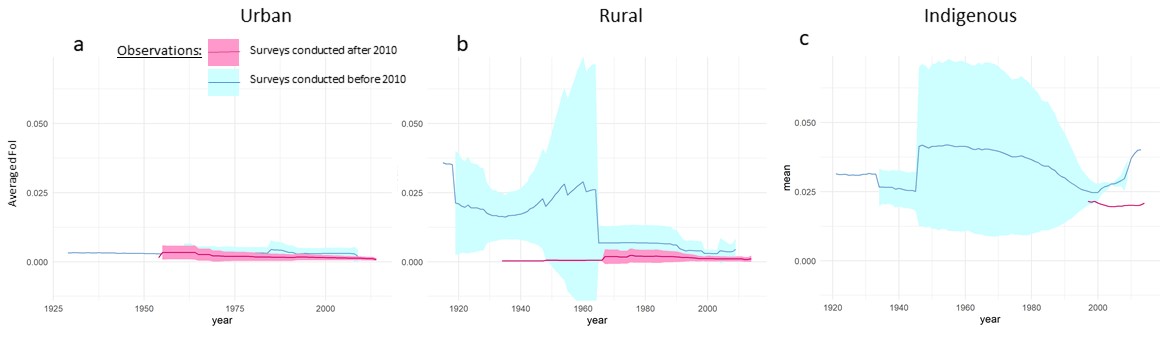


Supp. Figure 4 :Averaged observed FoI values by year for serosurveys conducted before 2010 in blue (n=39) and after or in 2010 in pink (n=40) for a) urban, b) rural and c) indigenous settings.

**Supplementary method**

*Models used to estimate the Force-of-Infection: Extracted from Z. M. Cucunubá, “Modelling the epidemiology and healthcare burden of Chagas disease in Colombia,” Imperial College of London. (2017)*

***“Catalytic Force-of-Infection Models***

*Descriptive prevalence results are reported as percentages and accompanied by 95% binomial (exact) confidence intervals (95% CI). For the force-of-infection models, we consider that if the rate of infection acquisition―here the rate of seroconversion―is constant over time, infection (sero)prevalence will increase monotonically with age as cumulative exposure increases. Formally,* $P_{a}= 1- exp(-{}_{a})$ *, with P_a_ the age-specific seroprevalence and λ the force-of-infection (the per susceptible incidence or FoI) as originally described by Muench, 1959 [9, 10]. More generally, the FoI may fluctuate over time t, modifying the seroprevalence age profiles. For a survey completed at time τ ,* $P_{a,}= 1- exp\left( -\int_{t=-a}^{t=} {}_{t}dt \right)$*. Therefore, a serosurvey completed at time τ , and including ages from {a_min_ , a_max_} , is informative on exposure (and FoI) between τ - a_max_ and τ . Other modelling assumptions included: a) no age-dependency in transmission [76], b) no seroreversion [76], and c) no specific migration due to Chagas infection status [11, 12].”*

*Based on the above, we used the posterior distribution of the FoI fitted with time-varying FoI (*${}_{i}$*) following:*

$$P_{a,}= 1- exp\left( -\sum_{i=-a=1}^{i=} {}_{i} \right)$$

**Supplementary References**

1. Z. M. Cucunubá, *et al.*, Modelling historical changes in the force-of-infection of Chagas disease to inform control and elimination programmes: application in Colombia. *BMJ Glob. Health* **2**, e000345 (2017).
2. Departamento Administrativo Nacional de Estadística (DANE): www.dane.gov.co (May 25, 2020).
3. H. E. Beck, *et al.*, Present and future Köppen-Geiger climate classification maps at 1-km resolution. *Sci. Data* **5**, 180214 (2018).
4. G. J. Parra-Henao, M. Flórez Martínez, V. M. Angulo Silva, Red Chagas Colombia, Vigilancia de Triatominae (Hemiptera: Reduviidae) en Colombia, 1era Edition., pp. 127 (Sic Editorial Ltda. 2013).
5. G. Parra-Henao, O. Quirós-Gómez, N. Jaramillo-O, Á. Segura Cardona, Environmental determinants of the distribution of Chagas disease vector *Triatoma dimidiata* in Colombia. *Am. J. Trop. Med. Hyg*. **94,** 767–774 (2016).
6. G. Parra-Henao, L. C. Suárez-Escudero, S. González-Caro, Potential distribution of Chagas disease vectors (Hemiptera, Reduviidae, Triatominae) in Colombia, based on ecological niche modeling. *J. Trop. Med*. **2016,** 1439090 (2016).
7. G. Parra-Henao, V. Angulo, Z. Cucunubá, Colombian Chagas Network. Final report, project 1. (2015).
8. Z. M. Cucunubá, “Modelling the epidemiology and healthcare burden of Chagas disease in Colombia,” Imperial College of London. (2017)
